# Characterization of single nucleotide polymorphism in the 5'-untranslated region (5'-UTR) of Lactoferrin gene and its association with reproductive parameters and uterine infection in dairy cattle

**Published:** 2012

**Authors:** Abolfazl Hajibemani, Hassan Sharifiyazdi, Abdolah Mirzaei, Abbas Rowshan Ghasrodashti

**Affiliations:** 1 *PhD Student at Department of Clinical Sciences, Faculty of Veterinary Medicine, Shiraz University, Shiraz, Iran; *; 2 *Department of Clinical Sciences, Faculty of Veterinary Medicine, Shiraz University, Shiraz, Iran; *; 3 *Department of Clinical Sciences, Faculty of Veterinary Medicine, Islamic Azad University, Kazerun Branch, Kazerun, Iran.*

**Keywords:** Lactoferrin, Polymorphism, Uterine infection, Dairy cow

## Abstract

Uterine infection is one of the reproductive diseases that can have disturbing postpartum uterine health in cattle. Therefore, identification of resistant genotypes to uterine infection is important. Lactoferrin (LF) is one of the major antimicrobial compounds in the normal uterine discharges of cows. We hypothesized that allelic diversity in LF gene may contribute to susceptibility or resistance to uterine infection. We investigated the single nucleotide polymorphism genes identified in the 5' untranslated region (5'-UTR, position +32) of the LF gene using Allele-specific PCR method in cows with and without uterine infection. Blood samples were collected from 89 multiparous Holstein dairy cows with a history of uterine infection (n = 51), and cows without disease as the control group (n= 38). The results indicated the presence of different proportion of polymorphisms (G > C) in the 5'-UTR area of cows in the all groups. The results of Allele specific PCR was in complete agreement with sequencing method. Statistical analysis did not show any statistically significant correlation between disease and SNP in 5'-UTR. While, there was a significant difference in the mean of reproductive parameters of cows without polymorphism compare to those of with SNP in 5'-UTR. Cows with +32:CC genotype and +32:GC genotype (cows with SNP in UTR) had lower average of services per conception and days open compared to cows with the +32:GG genotypes. However, no significant difference in the calving to first service was found between these genotypes. Further studies will be required to determine critical SNPs in LF gene and status of the risk of uterine infection and embryo survival in cows.

## Introduction

Uterine infection is an important disease affecting in the economic efficiency of dairy herds causing infertility.^1^ The presence of pathogenic bacteria in the uterus causes inflammation, delays uterine involution, and perturbs embryo survival.^2^ In addition, uterine bacterial infection, bacterial products suppress pituitary luteinizing hormone secretion and perturbs postpartum ovarian follicular growth and function disrupting ovulation in dairy cattle.^3^ Accordingly, uterine disease is associated with lower conception rate, increased intervals from calving to first service or conception, and more cattle culled for failure to conceive.^4^^-^^6^ The outcome of uterine infection depends on the number and virulence of the organisms present,^7^ as well as the condition of the uterus and its inherent defense mechanism.^8^ The innate immune system functions as the first line of defense during uterine infection and therefore plays an important role in early recognition and elimination of invading microorganisms.^9^ Uterine epithelial cells produce a spectrum of antimicrobials including lactoferrin (LF), lysozymes, and complements, which enter uterine lumen providing increased protection against potential pathogens.^10^ Lactoferrin constitutes an important component of the innate immune system, with well-characterized antibacterial, antiviral, and immune modulatory properties.^11^ Bovine LF is a monomeric metal-binding glycoprotein synthesized as a 708-amino acid (aa) protein with a 19-aa signal peptide.^12^^,^^13^ Lactoferrin consists of two globular lobes, each of which contains one iron-binding site. These lobes, designated as the N- and C-lobes, represent the N- and C-terminal halves of the polypeptide.^14^

The two metal-binding sites of LF that lie between the two domains of each lobe are highly similar to each other^14^ and to the corresponding sites in human LF.^15^ Iron- binding occurs concomitantly with the binding of two bicarbonate anions that appear to play a prominent structural role.^14^^-^^16^ The presence of LF in the bovine reproductive system has previously been confirmed by immunohistochemical study.^17^

We have recently demonstrated that A to C trans-version within the TATA box of bovine LF gene maybe responsible for increasing rate of uterine infection in dairy cows. ^18^ However, other polymorphic sites such as 5'-UTR may also be associated with increased risk for uterine infection due to its important role in regulation of gene expression. The aim of this study was to identify the single nucleotide polymorphisms in the 5'-untranslated region (5'- UTR) of the LF gene using Allele-Specific PCR technique and its relationship with uterine infections as well as some reproductive parameters in Holstein dairy cows. 

## Materials and Methods

**Animals and sample collection. **This study was carried out on registered multiparous Iranian Holstein cows at the farm of Farzis milk and meat producing complex in Shiraz, Fars province, south of Iran. Shiraz is located at a latitude of 29° 38′ N and longitude 52° 36′ E. Its altitude is 1296 meters above sea level. The climatic condition is relatively rainy with mild winters and hot dry summers with the average temperature of 17 C ranging between 5 and 30 C.^19^ Cows were fed standard rations (total mixed ration) including mainly alfalfa, corn silage, beet pulp, cotton seed, soybean, corn and barley. The cows were milked three times daily with the use of a pipeline milking machine.

A total of 89 pluriparous Holstein dairy cows were selected with confirmed history of uterine infection in the previous lactation. Fifty one cows with uterine infection and 38 cows without uterine infection as the control are selected. Cows included in the study had no peripartum diseases (dystocia, retained placenta, clinical hypo-calcaemia and ketosis) in the previous lactation. Cows were considered as infected that have clinical history of vulva discharge or intrauterine infusion or uterine flushing. Blood samples (2 mL) were collected from each cow via caudal venipuncture into tubes containing EDTA as anticoagulant and then were dispatched to the laboratory and stored at -20 °C for subsequent DNA extraction.

**Extracted genomic DNA. **Samples were removed from the -20° C freezer and then were melt and vortexed for 10 seconds. DNA was extracted using a DNA isolation kit (MBST, Iran) according to the manufacturer’s instructions. Briefly, 50 microliters of each sample was lysed in 180 μL lysis buffer and the proteins were degraded with 20 μL proteinase K for 10 min at 55 °C. After addition of 360 μL binding buffer and incubation for 10 min at 70 °C, 270 μL ethanol (96.0%) was added to the solution and after vortexing, the complete volume was transferred to the MBST-column. The MBST-column was first centrifuged, and then washed twice with 500 μL washing-buffer. Finally, DNA was eluted from the carrier using 100µL Elution buffer. The DNA was quantified spectrophotometrically, and the integrity assessed via agarose gel electrophoresis (0. 8%). Extracted genomic DNA samples were stored at -20°C for subsequent analysis.

**Primers. **The single nucleotide polymorphisms (SNPs) in the 5' untranslated region (5'- UTR, position +32 from transcription start site) of LF promoter gene (G/G at LF +32) were genotyped using a new Allele-specific PCR method. In the present study, three primer pairs were used for PCR. In addition to gene specific primers LF cow (Lac-1, Lac-2),^20^ (LFR1-R , LFR1-F),^21^ specific primers of each allele (HajR2, HajF2). Based on the +32 area of 5'-UTR LF gene was designed using the software Primer Premier 5. All oligonucleotide primers used in this study were synthes-ized by CinnaGen Company in Iran. Main characteristics of the primers and expected length of produced fragment by each pairs of primers are presented in Table 1.

**Table 1:** Characteristics of the specific primers used in PCR

	**primer**	**(5`- 3`)** **Sequence**	**Purpose**	**Annealing Temperature (°C)**	**Length of Production**
**Primer-1 Combination**	LFR1-F LFR1-R	5' -GACAGCCTTTGGGCACTTAG-3' 5' -GGGTAGGACAGAAGCGACAG-3'	Sequencing	57.8	1068bp
**Primer-2 Combination**	HajF2 LFR1-R Lac-1 Lac-2	5' -TCGTTCCGGAGTCGCCCCAGGA**AG**-3' 5' -GGGTAGGACAGAAGCGACAG-3' 5' -GCCTCATGACAACTCCCACAC-3' 5' -CAGGTTGACACATCGGTTGAC-3'	G allel specific detection Internal control	65.0 - 68.0	408bp 301bp
**Primer-3 Combination**	HajR2 LFR1-F Lac-1 Lac-2	5' -GGGACGAAGAGCTTCATGGCTG**TG**-3' 5' -GACAGCCTTTGGGCACTTAG-3' 5' -GCCTCATGACAACTCCCACAC-3' 5' -CAGGTTGACACATCGGTTGAC-3'	C allel specific detection Internal control	65.0 - 68.0	709bp 301bp

The underlined bases are those modified from the original sequence to increase the specificity of the allele-specific PCR.

**Allele specific PCRs and Genotyping. **Primer combination 2 was used to identify G allele in 5'- UTR and contained primers HajF2, LFR1-R, Lac-1, Lac-2; primer combination 3 also contained four primers (primers HajR2, LFR1-F, Lac-1, Lac-2) for detection of C allele. The PCR reaction (25 μL) was performed in 10 mM Tris–HCl, pH 8.4, 50 mM KCl, 1.5 mM MgCl_2_, 250 μM of each dNTP, 10-20 pmol of each primer (Cinnagen Inc., Tehran, Iran), and 0.5 U *Taq *DNA polymerase (Fermentas; Glen Burnie, Maryland) using 2 μL of DNA extracted as template.

Amplification was performed with a Bio-Rad thermo-cycler (Bio-Rad Laboratories Inc., Hercules, CA, USA) by using a single denaturation step (5 min at 94 °C), followed by a 35-cycle program, with each cycle consisting of denaturation at 94 °C, for 1 min, annealing at 65-68 °C for 45 sec, and extension at 72 °C for 45 sec, with a final extension; a final extension step (72 °C for 7 min) was also used. The PCR products were analyzed by agarose gel electrophoresis (3.0%) after ethidium bromide staining and visualized under ultraviolet transillumination.

**Sequencing. **The validity of our designed Allele-specific PCR was verified by testing 21 individuals including three polymorfisms. Related PCR products generated by LFR1-F and LFR2-R primers (Table 1) were sequenced at a commercial laboratory (Macrogen, Seoul, South Korea) using capillary DNA analyzer (ABI 3730, Applied Biosystems, Foster City, California) after sequencing reactions with a Big Dye Terminator V3.1 Cycle Sequencing Kit (Applied Biosystems). Point mutations were screened by DNA sequence analysis for +32 site in 5'UTR after multiple alignment using ClustalW alignment option available in the MEGA 4 software. 

**Statistical analysis. **The results were analyzed to find out the relationship between the polymorphism within 5' UTR, position +32 of the LF promoter region and uterine infection. The results were statistically analyzed using chi-square test for independence. Three categories of different mutation types of control (without uterine infection) and patient (with single or more occurrence of uterine infection) groups were tested for significant differences by Pearson Chi-Square test for 2 2 contingency tables. Reproductive parameters [calving to first service interval, days open (DO) and service per conception (SPC)] were compared between cows with different types of polymorphism by Kruskal–Wallis test. All results were statistically analyzed at the *P* ≤ 0.05 level of confidence using the SPSS statistical software (Version 15.0, SPSS Inc., Chicago, Illinois). 

## Results

**Fig. 1:**
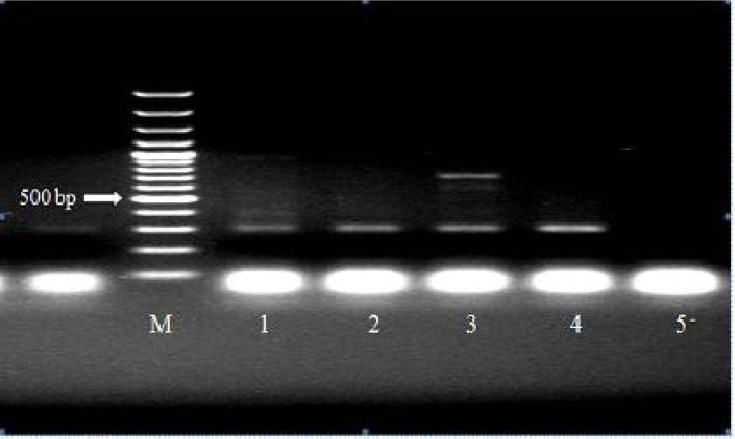
Electrophoresis pattern showing reaction PCR products of some samples. HajR2, LFR1-F and Lac1, Lac2 primers were used to amplify 709 bp fragment as major band (for C allele specific detection) and 301 bp fragment as internal control respectively. M: marker 100 bp. lane 1, 2: GG genotype. Lane 3: CC gnotype. Lane 4: Negative control (GG genotype). Lane 5: Distilled water.

**Fig. 2:**
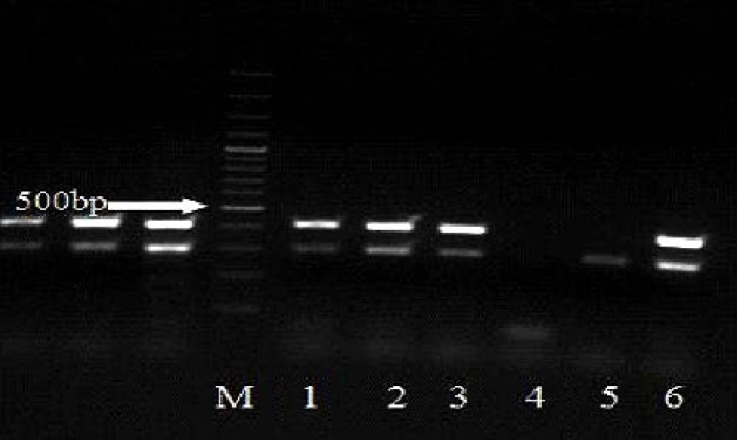
Electrophoresis pattern showing reaction PCR products of some samples. HajF2, LFR1-R and Lac1, Lac2 primers were used to amplify 408 bp fragment as major band (for G allele specific detection) and 301 bp fragment as internal control, respectively. M: marker 100 bp. lane 1: GC genotype; Lane 2, 3 and 6: GG genotype; Lane 4: Distilled water; Lane 5: CC genotype.

**Fig. 3:**
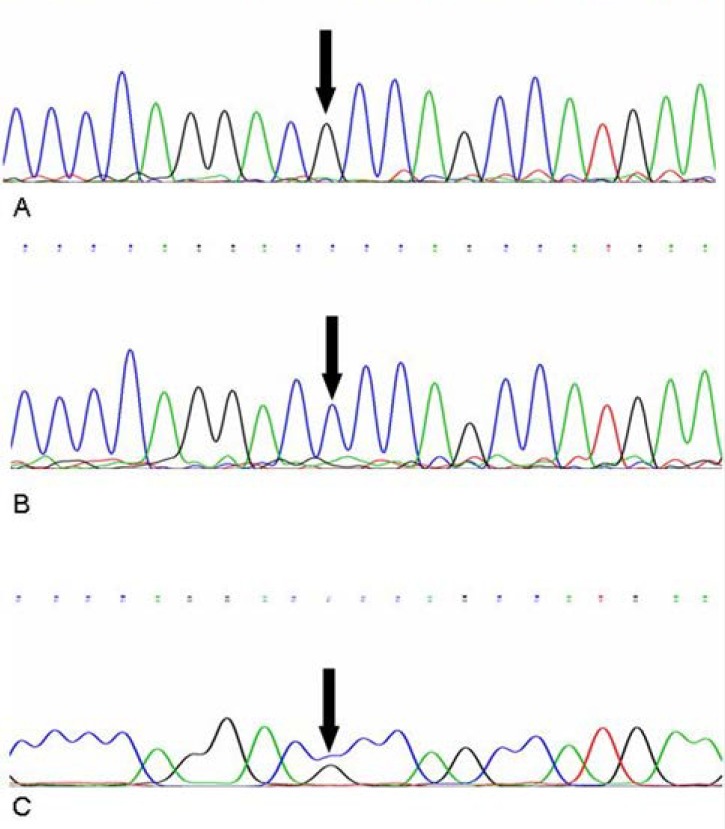
Partial chromatogram of the cow lactoferrin gene from different genotypes. A. Genotype GG. B. Genotype CC. C. Genotype GC. black thick arrow indicates point mutation within 5'-UTR, position +32).

As expected, Allele Specific PCR analysis of internal PCR products from different 5' UTR, position +32 genotypes (GG, GC, CC) showed three distinct banding patterns (Figs. 1 and 2). As the Allele Specific PCR method results were completely consistent with those of the direct sequencing method (Fig. 3). GG genotype showed no polymorphism in 5' UTR, position +32, GC genotype showed polymorphism in one alleles and CC genotype suggested that both alleles mutant in the position +32. Final characterization of each genotype performed based on the presence or absence of two bands of 408 bp and 709 bp. Accordingly, in the cows without mutation (+32:GG) and mutant homozygous cases (+32:CC), a fragment of 408 bp and 709 bp were amplified, respectively, while in heterozygous cases (+32:GC) both 408 bp and 709 bp fragments were produced (Figs. 1 and 2).

Surprisingly, in heterozygous samples (Lane 1:GC genotype), 408 bp fragment as major band was greatly reduced, and these samples showed weaker staining fragments than homozygous (GG genotype) in comparison with internal control band (301 bp). The weak staining of this fragment suggested reduced levels of this target DNA, which may be the reason for lower amplification. The results of the present study showed that the presence of polymorphism within 5'-UTR in the two groups. Cows with and without polymorphism within 5'-UTR, position +32 were compared regarding the mutation within 5'-UTR, position +32 in the two groups of with and without uterine infection (Table 2).

**Table 2:** Classification of 5'-UTR genotypes for cows with (n = 51) and without (n = 38) uterine infection based on the 5'-UTR Genotype (Position +32).

Uterine infection	5'-UTR Genotype (Position +32) %(N)			Total
	Without polymorphism (+32:GG)	polymorphism heterozygotes (+32:GC)	polymorphism homozygotes (+32:CC)	
Infected	64.70 (33)	21.56 (11)	13.72 (7)	100 (51)
Non-infected	52.63 (20)	36.84 (14)	10.52 (4)	100 (38)

N: number of cows

There were no significant differences between the occurrences of different polymorphism within 5'-UTR, position +32 for the two groups (*P* > 0.05). However, the occurrence of polymorphism homozygotes was lower compared to the polymorphism heterozygotes and without polymorphism of cows with uterine infection. 

Reproductive parameters were compared between cows with and without polymorphism within 5'-UTR, position +32. Significant differences were observed in the mean of reproductive parameters of cows without polymorphism compare to those of with SNP in UTR (Table 3). Cows with +32:CC genotype and +32:GC genotype (cows with SNP in UTR) had lower average of services per conception and days open compared to cows with the +32:GG genotypes (*P* <0.05). However, no significant difference in the calving to first service was found between these genotypes (*P *> 0.05). 

**Table 3:** Comparison of the reproductive parameters (Mean ± SD) between different UTR genotypes of dairy cows.

Reproductive parameters*	Genotype		
	Without polymorphism (+32:GG)	polymorphism heterozygotes (+32:GC)	polymorphism homozygotes (+32:CC)
Service per conception	1.81± 0.78^a^	1.69 ± 0.8^b^	1.40 ± 0.35^b^
Days open	118.70 ± 44.9^a^	98.80 ± 43.29^b^	78.90 ± 19.55^b^
Calving to first service	72.40 ± 18.01	66.30 ± 11.22	67.87 ± 12.37

* Data are the reproductive parameters for previous lactations of cows.

a,b Different superscript in rows indicate significant difference (*P* < 0.05).

## Discussion

Uterine infection is one of the most economically important diseases in the dairy industry. The outcome of uterine infection depends on the number and virulence of the organisms present,^7^ as well as the condition of the uterus and its inherent defense mechanism.^8^ The innate immune system constitutes the first line of defense against invading microbial pathogens during uterine infection.^9^ As previously described, LF is a multifunctional glycoprotein extensively investigated for its wide range of bioactivities including antimicrobial, antioxidant, immunostimulatory and anticancer effects.^22^^-^^26^

Preliminary studies were conducted to identify polymorphism in the gene promoter of the LF in different areas, revealed its association with mastitis and uterine infection in dairy cattle.^27^ Immunohistochemical study of the bovine reproductive system determined that LF is localized in the uterine and Bartholin's glands, the epithelial cells of the cervix and ampulla of the uterine tube.^17^ Northern blot analysis has shown that the LF gene is widely expressed in various bovine tissues.^17^^,^^28^ Accordingly, identification of the resistance genotypes to uterine infections in dairy herds based on LF gene (as a genetic resistance marker) and multiply them will reduce cost of treatment. 

Recently single nucleotide polymorphism (SNP) in the 5' untranslated region (5'-UTR, position +32) has been detected in dairy cows using direct sequencing. The polymorphism in position +32 was first discovered by Li *et al*. and is located adjacent to one of the putative Sp1 binding site, in exon 1 of 5' untranslated region and 7 bases upstream of the starting codon (ATG) in a near consensus Kozak sequence.^17^

In a study by Kaminiski *et al*., 358 Polish Holstein cows were screened by the SSCP method giving the percentage of genotypes 62.0%, 31.0% and 5.9% for GG, GC, and CC, respectively in position +32.^29^ Lactoferrin allele C has double positive effect in milk: increases protein yield and probably decreases SCC. They reported that the CC genotype had highest and lowest percentage of milk protein and SCC, respectively. However, *no* statistically *significant* correlations were found between CC genotype and SCC. Untranslated region contains important information that on one hand plays a role on the stability of mature mRNA and on other hand has important function in regulation of translation value and protein synthesis with a mechanism of Post-transcriptional regulation of gene expression.^30^

5'-UTR can also contain binding sites for transacting proteins, which can also modify the efficiency of mRNA translation. It is therefore thought that de-regulation of translation, via these 5'-UTR sequences, is responsible for a significant reduction in LF expression and that plays a key role in uterine infections. GC-rich region within 5'-UTR provided a capacity for mRNA to form stable stem- loop structure proximal to the 5'-cap.^31^ In addition, the importance of 5′UTR SNP for translational regulation has been demonstrated in several disease in human.^31^^-^^33^

 Different approaches have been used for SNP genotyping.^34^ Each methodology has advantages and disadvantages. Some of them being simple to use and fast; others requiring a considerable amount of time and labor, including multiple steps (like the PCR-RFLP) or complex and expensive equipment (like the mass spectroscopy). Thus, depending on the application and budget, the particular selection may vary.^35^ The allele-specific PCR is becoming a popular method for DNA typing of SNPs using different detection methods (Ethidium Bromide, Sybr Green, High-resolution LCGreen, etc.). The single-tube PCR genotyping does not require expensive or excessively complex equipment. In this work; we have optimized a genotyping procedure based on the use of allele-specific PCR. Such approach has been usually performed carrying out two independent PCR reactions of the same sample, priming each one of them with the appropriate allele-specific primer. After the amplification, the PCR products are segregated by agar gel electrophoresis and the results are analyzed.^36^ The validity of our rapid and economical method has been checked for three polymorphisms. The comparison of the results with those obtained using DNA sequencing has demonstrated the accuracy of our methodology. Although DNA sequencing may become the ultimate genotyping tool, it is currently expensive and time consuming compared to other alternatives.

In this study, we investigated the association between polymorphism in the 5' untranslated region (5'-UTR, position +32) and postpartum uterine infection and some reproductive performance of Holstein dairy cattle using Allele Specific PCR method in Iran. We did not find evidence for a significant association between SNP in the 5'-UTR (+32:G/G) LF gene and the uterine infection in dairy cows in Iran. While, there was a significant difference in the mean of reproductive parameters of cows without polymorphism compared to those of with mutation in 5'-UTR. Cows with +32:CC and +32:GC genotypes had lower average of services per conception and days open compared to cows with the +32:GG (wild-type) genotypes. However, there was no significant difference among these genotypes in the calving to first service. These findings support what has been assumed in earlier work that showed C allele increased gene expression of LF. ^29^ It seems likely that, the polymorphism in this area may cause innate immune system of uterus to increase. Therefore, it will be interesting to know whether these SNPs have any effect on the expression and biological function of LF. Recent studies have shown that the SNPs may be associated with many diseases and it is possible LF polymorphism could influence immunity and variations of bacterial resistance in different species.^18^^,^^32^^,^^33^

Regulation of LF gene expression has been previously reviewed by Teng Estrogen regulation of LF gene expression was also demonstrated in the uterus and vaginal epithelium of the rat and hamster.^37^ Patients with adenomatous hyperplasia of the uterus (chronically exposed to estrogen) persistently expressed LF gene in the endometrial epithelium suggesting a link between estrogen exposure and LF gene expression.^37^ It was found the expression of LF in pre-implantation mouse embryos^38^ accompanied with a high level of LF expression in preimplantation uterine epithelium in mouse.^39^

Lactoferrin may play an important physiological role in the uterus during the preimplantation period and early pregnancy.^38^ Since a large proportion of pregnancy losses occur in the preimplantation period, the interaction between early embryos and their maternal environment is vital for *embryo* development.^38^^,^^40^ It is not clear whether the preovulatory estrogen surge induces LF gene expression in the embryo and the uterine epithelium. Nonetheless, estrogen receptor has been detected in the embryo at this stage of development raising the possibility that estrogen also plays a direct role in the regulation of LF gene expression of the embryo.^37^

In conclusion, these findings suggest that G to C transversion within the 5'-UTR of bovine LF gene could partly be associated with an improvement on reproductive performance in dairy cows. It seems that the presence of this polymorphism in 5'-UTR of bovine LF gene may affect on the gene expression and somehow to decrease uterine infection resulting in higher reproductive performance. According to the antibacterial, antiviral, and immune modulatory properties of LF, further studies are needed to measure LF protein and mRNA levels in these three genotypes using ELISA and real-time RT-PCR, respectively.
